# Bicistronic Lentiviruses Containing a Viral 2A Cleavage Sequence Reliably Co-Express Two Proteins and Restore Vision to an Animal Model of LCA1

**DOI:** 10.1371/journal.pone.0020553

**Published:** 2011-05-27

**Authors:** Jonathan D. Verrier, Irina Madorsky, William E. Coggin, Mero Geesey, Michael Hochman, Elleanor Walling, Daniel Daroszewski, Kristofer S. Eccles, Rachel Ludlow, Susan L. Semple-Rowland

**Affiliations:** Department of Neuroscience, University of Florida McKnight Brain Institute, Gainesville, Florida, United States of America; The University of Hong Kong, Hong Kong

## Abstract

The disease processes underlying inherited retinal disease are complex and are not completely understood. Many of the corrective gene therapies designed to treat diseases linked to mutations in genes specifically expressed in photoreceptor cells restore function to these cells but fail to stop progression of the disease. There is growing consensus that effective treatments for these diseases will require delivery of multiple therapeutic proteins that will be selected to treat specific aspects of the disease process. The purpose of this study was to design a lentiviral transgene that reliably expresses all of the proteins it encodes and does so in a consistent manner among infected cells. We show, using both *in vitro* and *in vivo* analyses, that bicistronic lentiviral transgenes encoding two fluorescent proteins fused to a viral 2A-like cleavage peptide meet these expression criteria. To determine if this transgene design is suitable for therapeutic applications, we replaced one of the fluorescent protein genes with the gene encoding guanylate cyclase -1 (GC1) and delivered lentivirus carrying this transgene to the retinas of the GUCY1*B avian model of Leber congenital amaurosis – 1 (LCA1). GUCY1*B chickens carry a null mutation in the GC1 gene that disrupts photoreceptor function and causes blindness at hatching, a phenotype that closely matches that observed in humans with LCA1. We found that treatment of these animals with the 2A lentivector encoding GC1 restored vision to these animals as evidenced by the presence of optokinetic reflexes. We conclude that 2A-like peptides, with proper optimization, can be successfully incorporated into therapeutic vectors designed to deliver multiple proteins to neural retinal. These results highlight the potential of this vector design to serve as a platform for the development of combination therapies designed to enhance or prolong the benefits of corrective gene therapies.

## Introduction

Development of effective, long-lasting therapies for the treatment of progressive autosomal recessive retinal diseases that cause blindness early in life remains a challenge. Many of these diseases are caused by mutations in genes expressed exclusively in photoreceptor cells that disrupt their structure and function. There have been numerous studies showing that the effects of these mutant genes on photoreceptor cells can be reversed by delivering a normal copy of the mutated gene to these cells; however, in most cases these corrective gene therapies only provide a temporary reprieve from photoreceptor degeneration and the ensuing blindness that defines these diseases [1]–[3]. Because many of these aggressive photoreceptor diseases cause blindness early in life, it is desirable to develop treatment strategies that provide lifelong therapeutic benefits.

The most straightforward approach to achieving this treatment goal is to ensure that every photoreceptor in the diseased retina receives a copy of the corrective gene required to restore function to the cell before it has irreversibly committed itself to die. This strategy, while biologically sound, is currently unrealistic given the limitations of existing gene delivery methods. An alternate strategy to achieve this goal is suggested by examining the long-term therapeutic successes recently achieved using corrective gene therapy to treat Leber congenital amaurosis – 2 (LCA2) [4]–[7]. The gene mutated in LCA2 encodes retinal pigment epithelium-specific protein 65-kDa (RPE65), a protein that is specifically expressed in pigment epithelial cells and is critical for processing the vitamin A chromophore that photoreceptors need to regenerate their visual pigments following light stimulation [8], [9]. In the absence of this chromophore, photoreceptors are unable to respond to light and eventually degenerate [9]. In human retina, the ratio of retinal pigment epithelial cells to photoreceptor cells is approximately 1∶22 [10], one pigment epithelial cell supporting the function of about 22 photoreceptors. Thus, for every retinal pigment epithelial cell treated, approximately 22 photoreceptor cells regain function, a relationship that essentially amplifies the therapeutic benefits of the RPE65 therapy. In addition to amplifying the effect of RPE65 therapy, the relationship between the pigment epithelium and the adjacent photoreceptors also serves to minimize the number of untreated photoreceptor cells within treated areas which could positively influence the efficacy of the treatment if degeneration of untreated cells compromises survival of treated cells.

Unfortunately, unlike LCA2 therapies, the effects of corrective gene therapies designed to restore function to photoreceptors affected by genetic mutations located in photoreceptor genes are not amplified by retinal physiology or structure. Thus, developing methods to maximize the number of photoreceptor cells that receive corrective gene therapy remains a priority in developing treatments for these diseases. In addition to increasing the numbers of treated photoreceptors, can we prolong survival of these cells? One method to accomplish this would be to pair delivery of the corrective gene with delivery of additional therapeutic proteins that would enhance survival of photoreceptors. The results of our work on LCA1 have led us to consider this possible treatment route.

LCA1 is caused by null mutations in the GUCY2D gene (NC_000017.10) that encodes guanylate cyclase-1 (GC1), an enzyme that is expressed in photoreceptor cells [11]. GC1 plays a critical role in the ability of photoreceptor cells to recover from light stimulation [12] and its absence in humans [13] and in the GUCY1*B avian model of LCA1 [14] results in severely compromised vision or blindness at birth. We found that treatment of the retinas of these animals with a lentivirus carrying a normal copy of the GC1 gene restored function to the infected photoreceptor cells, as evidenced by measureable electroretinograms and visual behaviors including the optokinetic nystagmus (OKN) reflex [15]. The benefits of this therapy, which were clearly detectable in young animals, slowed but did not halt photoreceptor degeneration. We concluded from these results that corrective GC1 gene therapy is sufficient to restore function to photoreceptors lacking this enzyme and that, if we could prevent the treated photoreceptor population from degenerating, then we may be able to reach our current therapeutic goal of inducing lifelong sight in the GUCY1*B model of LCA1.

Over the past few years, we have been working to develop lentiviral-based, gene delivery strategies that would allow us to carry out meaningful studies of the effects of combination gene therapies on GUCY1*B photoreceptor function and survival. Ideally, the relative amounts of the proteins generated from these vectors should be highly consistent among infected cells. Our studies thus far have revealed that strategies involving delivery of either a mixture of two lentiviruses [16] or of lentiviruses carrying bicistronic transgenes constructed using a polio internal ribosome entry site (IRES) [15] fail to meet our protein expression criterion. Retinas treated with mixtures of two viruses contained very few infected cells that co-expressed the proteins encoded by the viruses in the mixture, a pattern that would be predicted if cells infected by one virus became refractory to additional infection [17]. In retinas treated with viruses carrying bicistronic IRES transgenes, the levels of protein produced from the cistron downstream of the polio IRES were often undetected in infected cells, a protein expression pattern that has been observed by others [18], [19]. We also examined the protein expression characteristics of lenitiviral transgenes carrying two internal promoters, each driving the expression of a single cistron [16], [20]. In general, both of the proteins encoded by the cistrons were detected in infected cells, but the relative amounts of these proteins among infected cells were highly variable.

In the present study, we sought to resolve the problems encountered with these approaches by 1) using two identical promoters in our dual-promoter lentivectors, 2) inserting an additional WPRE element between cistrons in our dual-promoter vectors, and 3) developing vectors carrying bicistronic transgenes containing the pTV1 viral 2A-like cleavage sequence. Viral 2A-like cleavage sequences, which were first identified in studies of the foot and mouth disease virus [21], are short peptide sequences that, when fused in frame between two cistrons, trigger co-translational ‘ribosomal skipping’, a process that produces equimolar quantities of the proteins encoded by the transcript. Inclusion of viral 2A-like cleavage sequences in viral transgenes has been shown to be an efficient approach for obtaining multiple, functional proteins from a single vector *in vitro* and *in vivo* [22]–[24]. Our results show that the protein expression characteristics of the 2A-containing vectors are superior to those obtained using the other approaches examined in this study in terms of protein level and the reproducibility of the protein expression pattern among infected cells. Importantly, we also show that GC1, delivered to GUCY1*B photoreceptors in the context of a 2A fusion peptide, is able to restore function to these cells as evidenced by the appearance of OKN reflexes in the treated animals. Based on these results, we conclude that 2A-like cleavage peptides, while not a panacea for all gene therapies, can, with proper optimization, be used to create bicistronic transgenes that reliably express both of their encoded proteins. Additionally, these peptides are small. For these reasons, they should prove to be useful in the context of many of the viral vectors currently being used in gene therapy applications.

## Results

### Design of Bicistronic Lentiviral Vectors

**Figure 1:**
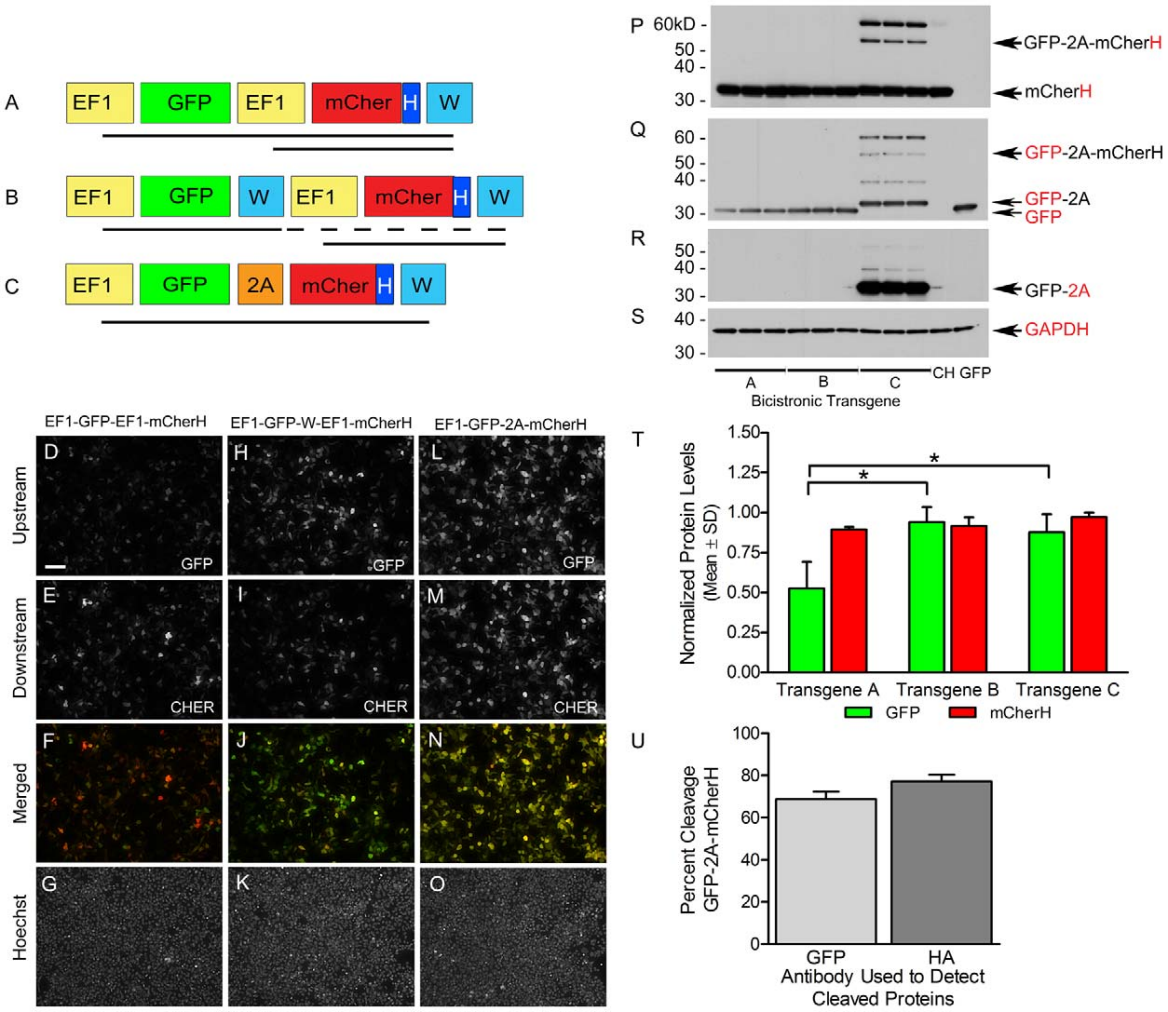
Evaluation of bicistronic transgene expression in transiently transfected HEK 293FT cell cultures. (**A–C**) Diagrams of the three bicistronic reporter transgenes analyzed in this study. The solid lines below each transgene indicate predicted mRNA transcripts. Abbreviations: W - WPRE; H - hemagglutinin tag; EF1 – elongation factor 1 promoter; GFP – green fluorescent protein; mCher – cherry fluorescent protein; 2A - porcine teschovirus (pTV1) 2A-like cleavage peptide. (**D–G**), (**H–K**) and (**L–O**) are representative images taken of HEK 293FT cell cultures that were transiently transfected with the lentivector plasmids carrying the indicated transgenes 48 h post-transfection. Image exposure times: GFP images in panels **D**,**H** and **L** = 17 ms; mCherH images in panels **E**, **I** and **M** = 4 ms; Hoechst images in panels **G**, **K** and **O** = 5 ms. Scale bar in panel D = 100 µm. (**P–S**) Western blot analyses of the expression of the GFP and mCherH reporter proteins encoded by the bicistronic transgenes. Three replicate HEK 293FT cultures were analyzed for each transgene and one culture was analyzed for each of the control vectors, EF1-mCherH (CH) and EF1-GFP (GFP). Four identical western blots containing the protein samples were generated and each was probed with an antibody recognizing either hemagglutinin (P), GFP (Q), 2A (R), or GAPDH (S). GAPDH was used as a protein loading control. (**T**) Densitometric analyses of GFP and mCherH protein levels produced from each transgene Significant differences (*p<.05) between the normalized mean GFP and mCherH values are indicated with brackets. (**U**) The cleavage efficiency of GFP-2A-mCherH was estimated by expressing the intensities of the cleaved GFP-2A (Q) or mCherH (P) protein bands as a percentage of total GFP or mCherH immunoreactivity (intensity of the cleaved plus uncleaved protein bands), respectively. Each estimate is the mean±SD of three samples.

Three different bicistronic transgenes, each encoding GFP and hemagglutinin (HA)-tagged mCherry (mCherH) fluorescent reporter proteins, were cloned into our pFIN lentivector backbone [20]. The first bicistronic transgene carries two identical internal EF1 promoters (pFIN-EF1-GFP-EF1-mCherH-WPRE, Figure 1A), each driving expression of one of the fluorescent proteins. This transgene was constructed with a promoter that is ubiquitously expressed and exhibits relatively high activity. It has been suggested that use of the same promoter in constructing dual-promoter vectors could lead to inadvertent DNA recombination during either viral packaging or processing of the viral RNA in the infected cells [25]; however, we did not see evidence of these problems when testing dual-promoter vectors carrying identical photoreceptor-specific promoters [16]. Thus, we chose to examine the performance of the EF1 dual-promoter transgene, a construct that would be useful in applications requiring higher protein expression levels. The second bicistronic transgene was constructed by inserting a second Woodchuck hepatitis posttranscriptional regulatory element (WPRE) between the upstream cistron and the second EF1 promoter of the dual EF1 promoter vector (pFIN-EF1-GFP-WPRE-EF1-mCherH-WPRE, Figure 1B). This modification has been shown to improve expression of dual-promoter vectors carrying two identical promoters [26]. Finally, the third bicistronic transgene was constructed using the well-characterized porcine *Teschovirus* (pTV1) 2A-like cleavage peptide. This peptide sequence produces two proteins from a single transcript by inducing co-translational “ribosomal skipping” [27]. During the “cleavage” process, 18 of the 19 amino acids comprising the 2A peptide, save the C-terminus proline, remain fused to the upstream peptide. The C-terminus proline remains attached to the N-terminus of the downstream peptide. This transgene was created by fusing the cDNAs encoding GFP and mCherH in frame with the pTV1(2A) sequence (pFIN-EF1-GFP-2A-mCherH-WPRE, Figure 1C).

### Expression of the Bicistronic Transgenes in Transiently Transfected HEK 293FT Cell Cultures

In this series of experiments, we examined the expression characteristics of the three bicistronic transgenes in transiently transfected HEK 293FT cells by monitoring expression of GFP and mCherH using both fluorescent microscopy and western blot. Fluorescent imaging of live cells was used to determine if GFP and mCherH could be detected in transfected cells and if the relative amounts of these two proteins were uniform among transfected cells. Comparisons of GFP (Figure 1D,H,L) and mCherH (Figure 1E,I,M) expression in transfected cultures indicated that the levels of GFP in cultured cells expressing the EF1-GFP-EF1-mCherH transgene were significantly lower than those expressing the other two transgenes while expression of mCherH from all three transgenes appeared to be similar. Examination of the merged GFP and mCherH images (Figure 1F,J,N) revealed that expression of GFP and mCherH was most uniform across cells in cultures expressing the EF1-GFP-2A-mCherH transgene; nearly all of the transfected cells appeared yellow (Figure 1N). In cultures expressing the EF1-GFP-W-EF1-mCherH transgene, expression of GFP and mCherH across cells was moderately uniform; transfected cells were either green or yellow (Figure 1J). Expression of GFP and mCherH was least uniform across cells in cultures expressing the EF1-GFP-EF1-mCherH; in these cultures, transfected cells ranged from green to red (Figure 1F).

The expression of each transgene was also analyzed using western blot (Figure 1P-S). Blots were prepared in quadruplicate and were probed with antibodies against either the HA tag (Figure 1P), GFP (Figure 1Q), 2A (Figure 1R) or glyceraldehydes 3-phosphate dehydrogenase (GAPDH) (Figure 1S). Examination of the blots probed with either the HA tag or the GFP antibody showed that each antibody specifically recognized either mCherH or GFP, respectively, in protein samples prepared from cultures expressing either the EF1-GFP-EF1-mCherH or the EF1-GFP-W-EF1-mCherH transgene. In samples isolated from cultures expressing the EF1-GFP-2A-mCherH transgene, these antibodies detected both cleaved and uncleaved GFP-2A-mCherH. Cleaved GFP-2A was also readily detected using the 2A antibody, which has been reported to bind to 2A in the context of the cleaved peptide but not in the context of uncleaved peptide [28]. The relative amounts of GFP and mCherH produced from each transgene were determined using a scanning densitometer and the GFP and mCherH values were normalized to the highest value obtained for each protein, respectively (Figure 1T). Uncleaved GFP-2A-mCherH was not included in the determination of the amount of GFP and mCherH produced in cells expressing the EF1-GFP-2A-mCherH transgene. An analyses of variance revealed significant differences between transgenes in terms of GFP expression (F(2,6)  = 8.92, p = 0.016); post hoc analyses using Tukey tests showed that the relative amount of GFP produced by the EF1-GFP-EF1-mCherH transgene (M = 0.53, SD = 0.17) was significantly lower than that produced from either the EF1-GFP-WPRE-EF1-mCherH (M = 0.94, SD = 0.10) or the EF1-GFP-2A-mCherH (M = 0.88, SD = 0.11) transgene. No differences were observed in relative mCherH expression between transgenes (F(2,6)  = 3.515, p = 0.098). Estimates of the cleavage efficiency of GFP-2A-mCherH were calculated by expressing the intensities of the cleaved mCherH (Figure 1P) or GFP-2A (Figure 1Q) protein bands as a percentage of total HA-tag or GFP immunoreactivity, respectively. The estimates produced from these calculations were 69%±4 (GFP, M±SD; n = 3) and 77%±3 (HA, M±SD; n = 3). Thus, conservatively the cleavage efficiency of the GFP-2A-mCherH protein expressed in the HEK 293FT cells was approximately 73% (Figure 1U).

### Expression of the Packaged Viral Bicistronic Transgenes in 293FT Cell Cultures

**Figure 2:**
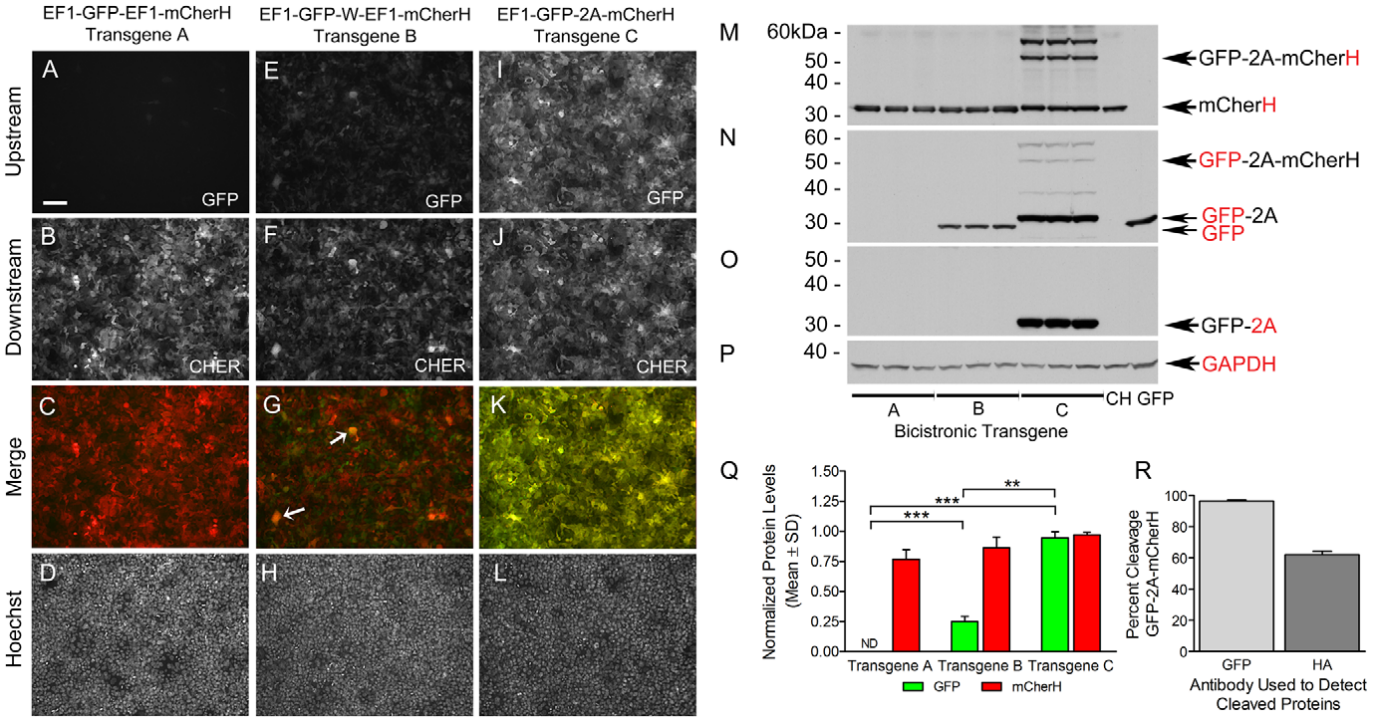
Expression characteristics of lentiviruses carrying the bicistronic transgenes in transduced HEK 293FT cells. (**A–D**), (**E–H**) and (**I–L**) are representative images of HEK 293FT cell cultures transduced with the indicated lentiviruses 96 h post-transduction. Image exposure times: GFP images in panels A, E, and I = 500 ms; mCher images in panels B, F, and J = 180 ms; Hoechst images in panels D, H, and L = 5 ms. Scale bar in A = 100 µm. (**M–P**) Western blot analyses of the expression of the GFP and mCherH reporter proteins in HEK293FT cells transduced with the lentiviruses. Three replicate HEK 293FT cultures were analyzed for each virus and one culture was analyzed for each of the control viruses, EF1-mCherH (CH) and EF1-GFP (GFP). Four identical western blots containing the protein samples were generated and each was probed with an antibody recognizing either hemagglutinin (M), GFP (N), 2A (O), or GAPDH (P). GAPDH was used as a protein loading control. (**Q**) Densitometric analyses of GFP and mCherH protein levels produced from each transgene. Normalized mean GFP and mCherH values were compared using paired T-tests and significant differences between samples (**p<.01, ***p<.001) are indicated with brackets. (**R**) The cleavage efficiency of GFP-2A-mCherH was estimated by expressing the intensities of the cleaved GFP-2A (N) or mCherH (M) protein bands as a percentage of total GFP or mCherH immunoreactivity (intensity of the cleaved plus uncleaved protein bands), respectively. Each estimate is the mean±SD of three samples.

This series of experiments was carried out to determine if viral delivery of the bicistronic transgenes alters the expression characteristics of the transgenes. HEK 293FT cell cultures were infected with lentiviruses that carried one of the three bicistronic transgenes and expression of GFP and mCherH were analyzed using fluorescent microscopy and western blot. Comparisons of cultures infected with these viruses showed that the levels of GFP produced from the EF1-GFP-EF1-mCherH (Figure 2A) and EF1-GFP-W-EF1-mCherH (Figure 2E) transgenes were significantly lower than those produced from the EF1-GFP-2A-mCherH transgene (Figure 2I). Relative levels of mCherH produced from all three transgenes were similar (Figure 2B, F, J). Merged GFP and mCherH images (Figure 2C, G, K) were examined to determine if the levels of expression of these proteins was uniform across infected cells. Cells infected with pFIN-EF1-GFP-EF1-mCherH-WPRE virus contained high levels of mCherH and very little to no detectable GFP (Figure 2C), while those infected with pFIN-EF1-GFP-2A-mCherH-WPRE virus contained high levels of both proteins. The few cells infected with pFIN-EF1-GFP-EF1-mCherH-WPRE virus that did contain trace amounts of GFP did not appear to express mCherH. Expression of GFP and mCherH was the least uniform in cultures transduced with pFIN-EF1-GFP-W-EF1-mCherH-WPRE virus (Figure 2G). The majority of the cells in these cultures expressed either GFP or mCherH; however, a subpopulation of the cells was present that co-expressed GFP and mCherH as evidenced by their yellow hue in the merged image (Figure 2G, arrows).

The expression of each transgene was also analyzed using western blot (Figure 2M–P). Blots were prepared in quadruplicate and were probed with antibodies against either the HA tag (Figure 2M), GFP (Figure 2N), 2A (Figure 2O) or GAPDH (Figure 2P). The relative amounts of GFP and mCherH produced from each transgene were determined using a scanning densitometer and the values were normalized to the highest value obtained for each protein, respectively (Figure 2Q). Uncleaved GFP-2A-mCherH was not included in the determination of the amount of GFP and mCherH produced in cells expressing the EF1-GFP-2A-mCherH transgene. An analyses of variance revealed significant differences between transgenes in terms of the amount of GFP expressed (F(2,6)  = 315.13, p<0.001); post hoc analyses using Tukey tests showed that the level of GFP produced by EF1-GFP-W-EF1-mCherH (M = 0.25, SD = 0.05) was significantly lower than that produced by EF1-GFP-2A-mCherH (M = 0.95, SD = 0.06). The levels of GFP produced by EF1-GFP-EF1-mCherH were not detectable on western blots but very low levels of this protein could be detected in a small minority of the transduced cultured cells (Figure 2A). No differences were observed in the levels of mCherH produced by the three transgenes (F(2,6)  = 4.22, p = 0.072). Estimates of the cleavage efficiency of GFP-2A-mCherH were calculated by expressing the intensities of the cleaved mCherH (Figure 2M) or GFP-2A (Figure 2N) protein bands as a percentage of total HA or GFP immunoreactivity, respectively. The estimates produced from these calculations were 96%±0.6 (GFP, M±SD; n = 3) and 62%±2.3 (HA, M±SD; n = 3) (Figure 2R).

### Expression of the Packaged Viral Bicistronic Transgenes in Chicken Retina

**Figure 3:**
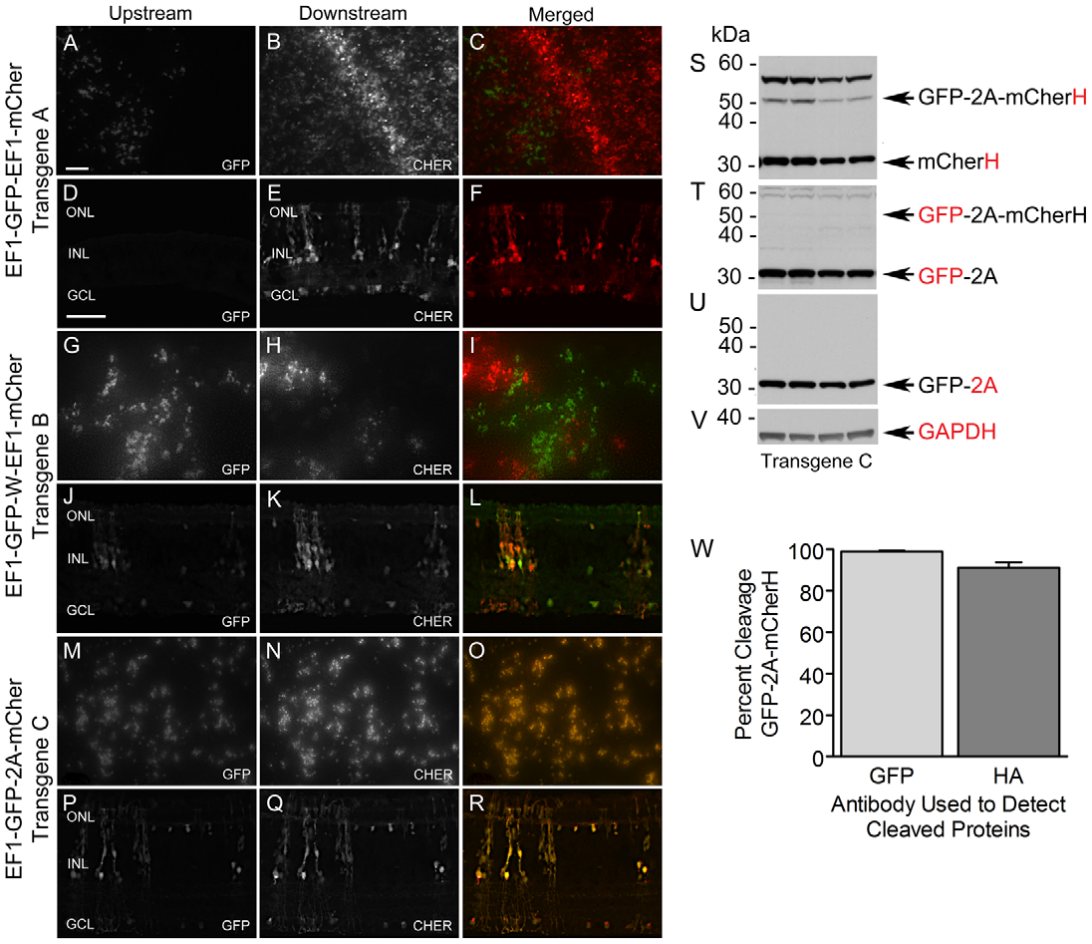
Expression characteristics of bicistronic lentiviruses in chicken retina. Lentiviruses were injected and retinal whole mounts and sections were imaged as described in the methods. (**A–C**) Whole mount and (**D–F**) representative section of a retina treated with pFIN-EF1-GFP-EF1-mCher-WPRE virus show robust expression of the downstream reporter (mCher). The upstream reporter (GFP) was either weakly expressed (A) or not detected (D). (**G–I**) Whole mount and (**J–L**) representative section of a retina treated with pFIN-EF1-GFP-WPRE-EF1-mCher-WPRE virus revealed that both reporter proteins were expressed in the treated retina but that only one of the reporter proteins could be detected in the majority of cells expressing the transgene. (**M-O**) Whole mount and (**P–R**) representative section of a retina treated with pFIN-EF1-GFP-2A-mCher-WPRE show robust co-expression of both reporter proteins in transduced cells. Scale bars in A and D = 50 µm. (**S–V**) Western blot analyses of the retinas of four chickens treated with pFIN-EF1-GFP-2A-mCher(H)-WPRE virus. Four replicate blots containing proteins extracted from the retinas were probed with antibodies to either hemagglutinin (S), GFP (T), 2A (U), or GAPDH (V). (**W**) The cleavage efficiency of GFP-2A-mCherH was estimated by expressing the intensities of the cleaved GFP-2A (T) or mCherH (S) protein bands as a percentage of total GFP or mCherH immunoreactivity (intensity of the cleaved plus uncleaved protein bands), respectively. Each estimate is the mean±SD of three samples.

In this series of experiments, the three bicistronic lentiviruses were injected into chicken embryos to determine if their expression characteristics in neural retina were similar to those observed in HEK 293FT cells. Retinal whole mounts and cryosections were examined using fluorescent microscopy to determine if GFP and mCherH could be detected in infected cells and if the relative amounts of these proteins were uniform among these cells. Examination of whole mounts (Figure 3A–C) and sections (Figure 3D–F) of retinas transduced with pFIN-EF1-GFP-EF1-mCher-WPRE revealed that the relative expression levels of mCherH were much higher than GFP in these retinas; the few cells that expressed detectable levels of GFP did not appear to be co-expressing mCher. Both reporter proteins were detected in retinas transduced with pFIN-EF1-GFP-WPRE-EF1-mCher-WPRE; however, the vast majority of the cells expressing this bicistronic transgene were positive for either GFP or mCher (Figure 3G–I). We were able to identify scattered groups of cells in retinal sections that expressed both reporter proteins, but they were rare and the relative amounts of each protein in these cells were highly variable (Figure 3J–L). Finally, examination of retinas transduced with the pFIN-EF1-GFP-2A-mCher-WPRE virus showed that both of the reporter proteins encoded by this virus were robustly expressed in these retinas (Figure 3M–R) and that the relative amounts of these proteins in infected cells were similar. Together, these results indicate that the expression characteristics of these three viruses are similar in HEK 293FT and chicken neural retinal cells.

The variability in the percent of the total area of the retinas of embryos transduced by the bicistronic viruses precluded the use of western blot analyses of homogenates of these retinas to determine the relative expression levels of GFP and mCherH generated from each of the viral transgenes. However, we were able to examine the cleavage efficiency of the GFP-2A-mCherH peptide within individual retinas transduced with pFIN-EF1a-GFP-2A-mCherH-WPRE virus using western blot (Figure 3S–V). Blots containing proteins isolated from the retinas of four chickens that had been treated with pFIN-EF1a-GFP-2A-mCherH-WPRE virus and one untreated control retina (WT) were probed with antibodies recognizing either the HA tag (Figure 3S), GFP (Figure 3T), 2A (Figure 3U) or GAPDH (Figure 3V). Examination of the HA tag and GFP staining patterns suggested that cleavage of the GFP-2A-mCherH peptides in infected retinal cells was efficient, results that were corroborated by the 2A staining pattern (Figure 3U). Estimates of the cleavage efficiency of GFP-2A-mCherH were calculated by expressing the intensities of the cleaved mCherH (Figure 3S) or GFP-2A (Figure 3T) protein bands as a percentage of total HA or GFP immunoreactivity, respectively (Figure 3W). The estimates produced from these calculations were 99%±0.4 (GFP, M±SD; n = 4) and 91%±2.6 (HA, M±SD; n = 4). These data show that our bicistronic transgene carrying a 2A cleavage sequence can be properly transcribed and translated in retinal cells *in vivo*.

### Expression of 2A Bicistronic Transgenes Encoding Guanylate Cyclase-1 in Transiently Transfected HEK 293FT Cell Cultures

**Figure 4:**
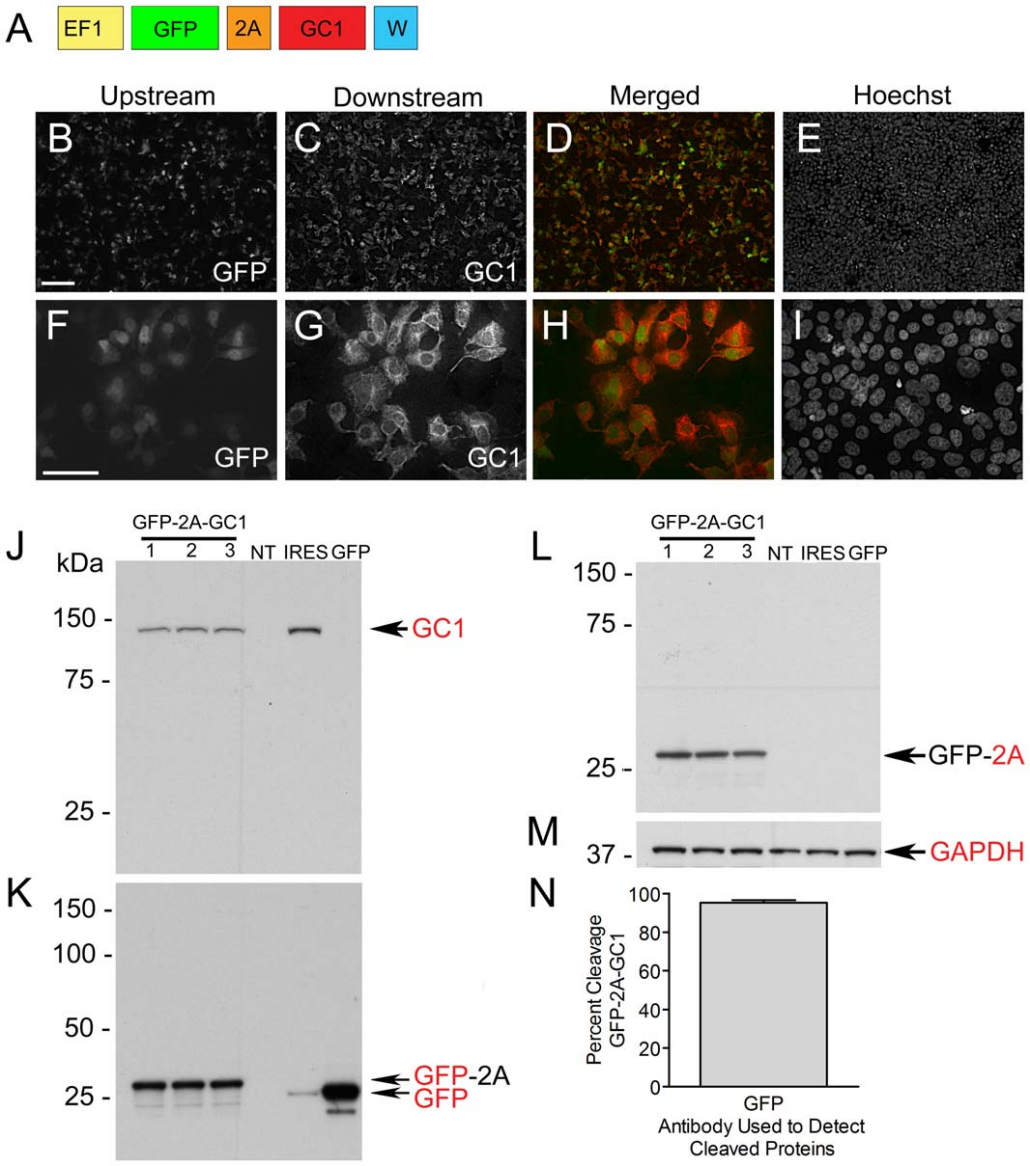
Evaluation of the expression characteristics of the EF1-GFP-2A-GC1 transgene in transiently transfected HEK 293FT cells. (**A**) Diagram of the EF1-GFP-2A-GC1 transgene. (**B–E and F–I**) Representative images of transiently transfected HEK 293FT cell cultures expressing EF1-GFP-2A-GC1 imaged 48 h post-transfection. Scale bars shown in B and F equal 100 and 50 µm, respectively. (**J–M**) Western blot analyses of HEK 293FT cells transiently transfected with pFIN-EF1-GFP-2A-GC1-WPRE (n = 3) probed for GC1 (J), GFP (K), 2A (L), and GAPDH (M). Non-transfected HEK 293FT cells (NT), HEK 293FT cells transfected with pFIN-EF1-GC1-IRES-GFP (IRES), and HEK 293FT cells transfected with pFIN-EF1-GFP-WPRE served as controls. GAPDH served as a loading control. (**N**) The cleavage efficiency of GFP-2A-GC1 was estimated by expressing the intensities of the cleaved GFP-2A (K) protein bands as a percentage of total GFP immunoreactivity (intensity of the cleaved plus uncleaved protein bands). The estimate is the mean±SD of three samples.

Prompted by the excellent expression characteristics exhibited by the 2A containing reporter lentiviruses *in vitro* and *in vivo*, we constructed a therapeutic 2A vector encoding guanylate cyclase -1 (GC1) and examined its expression in transiently transfected HEK 293FT cells. The transgene we constructed encoded GFP, which was used to identify transfected or transduced cells, and the gene encoding GC1that was fused in frame to the 2A peptide downstream of GFP (Figure 4A). GC1 is a transmembrane protein that is normally trafficked to the photoreceptor outer segments where it plays a critical role in recovery of these cells from light stimulation [12]. We have previously shown that delivery of bovine GC1 to the photoreceptors of the GUCY1*B chicken model of LCA1 in the context of an IRES-containing lentiviral transgene (pTYF-EF1-GC1-IRES-GFP) temporarily reversed blindness in these animals [15]. In this experiment, we evaluated the expression of EF1-GFP-2A-GC1 in transiently transfected 293FT cells using fluorescent microscopy and western blot.

Examination of HEK 293FT cell cultures transfected with pFIN-EF1-GFP-2A-GC1-WPRE DNA (Figure 4B–I) revealed that all transfected cells expressed GFP and GC1. In normal photoreceptor cells, GC1 is localized to the membranes of the outer segments. Thus, we expected that the GC1 produced from the GFP-2A-GC1 peptide would localize to the membranes of the transfected cells, which was found to be the case. Immunostained GC1 was localized primarily to the cell membranes (Figure 4G,H) while GFP filled the cell bodies (Figure 4F,H), locations consistent with the expected subcellular trafficking of these proteins.

Western blot analyses of cells transfected with pFIN-EF1-GFP-2A-GC1-WPRE showed that the cell lysates contained cleaved GC1 (Figure 4J) and GFP-2A (Figure 4K,L). Uncleaved GFP-2A-GC1 peptide was detected but only when the blots probed with the GFP antibody were highly overexposed. Quantification of the cleavage efficiency of the GFP-2A-GC1 peptide revealed that it was 95.3%±1.3 (GFP, M±SD; n = 3) (Figure 4N).

### Evaluation of the Therapeutic Potential of pFIN-EF1-GFP-2A-GC1-WPRE

**Figure 5:**
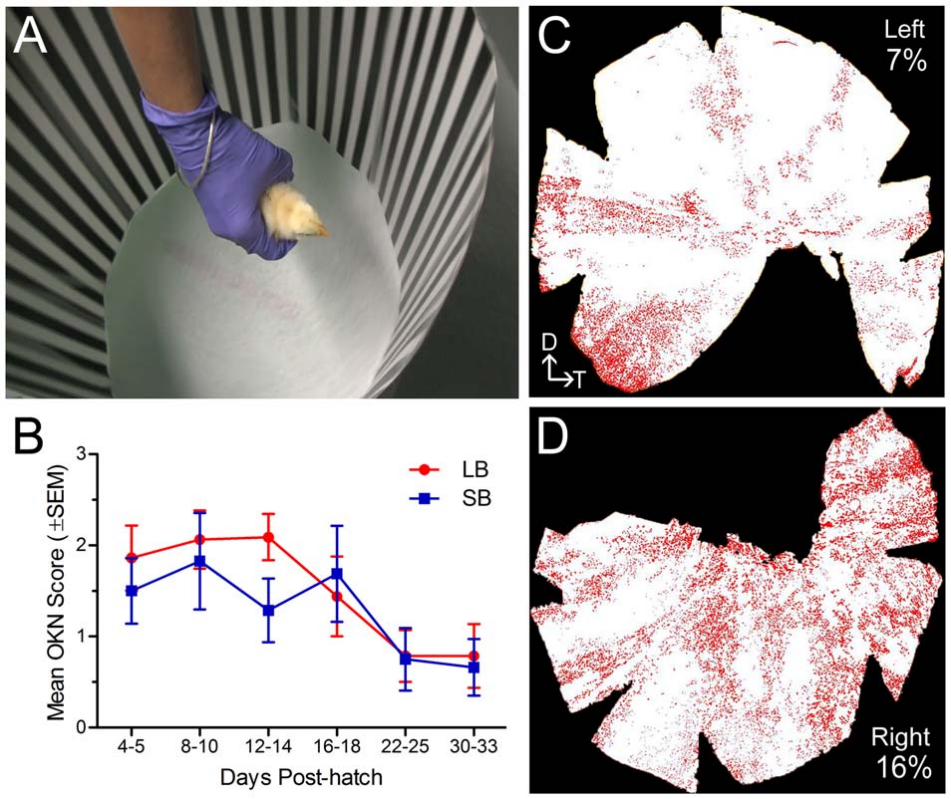
Analyses of the optokinetic (OKN) reflex responses of GUCY1*B chickens treated with pFIN-EF1-GFP-2A-GC1-WPRE virus. (**A**) Frame of OKN video showing the optokinetic testing apparatus. Videos of the OKN responses obtained from wild-type (Video S1), untreated GUCY1*B (Video S2) and treated GUCY1*B chickens (Video S3) are provided as supplemental data. (**B**) Summary of OKN responses obtained from seven treated GUCY1*B animals. The plotted values are the means±SEM of the average OKN responses obtained in response to either the large bar (LB) or small bar (SB) stimuli. (**C, D**) Whole mounts of the left (C) and right (D) retinas of a 5-week-old animal that had been treated with the virus. The percentage of the total retinal area infected by the virus was estimated using ImageJ software. The orientation of the retinas (D-dorsal; T-temporal) is indicated in panel C.

In our final experiment, we set out to determine if the integrated EF1-GFP-2A-GC1 transgene would produce sufficient levels of GC1 to restore function to the photoreceptors of GUCY1*B chickens. To be effective, the 2A fusion peptide expressed in photoreceptors needed to be cleaved efficiently and the resulting GC1 enzyme had to be properly trafficked to the outer segments of the photoreceptors. Stage 8–10 GUCY1*B embryos were injected with pFIN-EF1-GFP-2A-GC1-WPRE, hatched, and tested weekly for the presence of the optokinetic visual response (OKN) until they reached 5 weeks-of-age. The OKN responses of 7 treated animals were evaluated once a week for five weeks by holding the animals in the center of rotating platform that supported high contrast, visual stimuli consisting of vertical square wave gratings with spatial frequencies of 0.065 or 0.26 cycles·degree^−1^ that corresponded to bar widths of 5 or 1.25 cm, respectively (Figure 5A). These responses were compared to those obtained from wild-type and untreated GUCY1*B animals. OKN responses are driven primarily by the peripheral regions of the retina [29], [30], and unlike mammalian OKN responses, these responses in chickens are monocularly driven, the responses to clock-wise and counter-clockwise rotation of the visual stimuli being driven by the left and right eyes, respectively [31]. The OKN responses of the wild-type chickens were robust, characterized by well-defined lateral head movements synchronized with the speed of rotation of the visual stimulus (Video S1), while the untreated GUCY1*B chickens failed to respond to the stimuli (Video S2). All of the treated embryos that hatched produced OKN responses (Figure 5B; Video S3). A repeated measures ANOVA was used to examine the effects of stimulus type on the magnitude of the OKN responses over time. The results indicated that the response magnitude significantly decreased over the course of the 5-week test period (F(5,60)  = 5.0; p = 0.0007), in the absence of a main effect of stimulus type.

At the end of the 5-week testing period, we analyzed whole mounts of treated animal's retinas to obtain an estimate of the efficiency of our viral treatment. Our viral delivery method leads to infection of progenitor cells in the developing neural tube, some of which generate the neural retina. Examples of the viral transduction patterns produced in the retinas of our treated animals are shown in Figure 5 (C,D). Unlike the transduction pattern produced following subretinal delivery of viral vectors that is characterized by one large, contiguous population of infected cells, the transduction pattern produced following neural tube delivery of viral vectors is characterized by numerous scattered groups of infected cells that are distributed across the entire retina. The estimates of the total area transduced by the virus in the retinas shown in Figure 5 were 7% and 16%. These retinas belonged to the animal in the treated group whose OKN responses were assigned a rating of 3 throughout the entire 5-week test period. The estimated total area transduced in the retinas of animals whose OKN responses declined over the test period ranged from 3–7%. The strength and stability of these responses over time were very similar to those that we observed in animals treated with our IRES GC1 bicistronic vectors [15]. Overall, the results of the current study are encouraging because they show that the GC1 encoded by the 2A transgene is functional and that visual responses can be generated by a relatively small number of functioning photoreceptor cells. Importantly, they also suggest that relatively small increases in the total number of transduced photoreceptors in treated retinas could significantly increase the strength and stability of visual responses in treated animals.

## Discussion

The results of this study clearly show that it is possible to obtain consistent expression of two proteins from viral transgenes consisting of two cistrons joined in frame by the pTV1 viral 2A-like cleavage sequence. Importantly, the results of our study establish the therapeutic potential of this specific vector design. We found that we were able to reverse blindness in GUCY1*B chickens by treating them with pFIN-EF1-GFP-2A-GC1-WPRE virus, a finding that indicates that the GC1 encoded by this transgene was properly processed and was expressed in sufficient amounts in photoreceptor cells expressing the transgene to support the function of these cells. Together, these results highlight the potential of this vector design to serve as a platform for development of combination therapies designed to enhance or prolong the benefits of corrective gene therapies.

The cleavage characteristics and small sizes of 2A-like cleavage sequences make them particularly useful in developing multicistronic viral vectors [22]. Since the proteins encoded by transgenes containing 2A sequences are translated from one transcript, co-translational cleavage of these polypeptides is expected to produce equimolar quantities of the proteins [32]–[34]; however, proper cleavage and post-translational processing of the proteins does not always occur as expected. The cleavage efficiency of the polypeptide can be influenced by the nucleotide sequences flanking 2A and the order of the cistrons in the transgene relative to the 2A sequence [23], [28], [35]–[37]. The order of the cistrons relative to the 2A sequence can also alter post-translational processing and intracellular trafficking of the proteins encoded by the cistrons [28], [36], [37]. The small size of the 2A sequence is a second advantage of this technology, a feature that is particularly attractive when constructing multicistronic transgenes using viral vectors with limited cargo capacity.

Two counter indications to using 2A peptides are poor cleavage efficiency of the translated polypeptide and disruption of function of the upstream protein by the residual 2A peptide that remains fused to its C-terminus. Poor cleavage efficiency could lead to accumulation of significant amounts of uncleaved protein and the formation of toxic protein aggregates in cells expressing the transgene. Strategies that have been reported to significantly improve cleavage efficiency of 2A polypeptides include insertion of either a furin protease cleavage site or a furin site plus an amino acid spacer upstream of the GSG linker- 2A sequence [38], [39]. To determine if addition of a furin site to our EF1-GFP-2A-mCher transgene could increase cleavage of the 2A polypeptide, we constructed and compared the cleavage efficiency of GFP-furin-2A-mCher to that of GFP-2A-mCher. EF1-GFP-Furin-2A-mCher was constructed by inserting nucleotides encoding furin binding site (RAKR) upstream of the GSG-2A sequence. Western blot analyses of HEK 293FT cells expressing these transgenes revealed that addition of the furin site interfered with 2A cleavage (Figure S1). Very little of the polypeptide translated from the EF1-GFP-Furin-2A-mChe transgene was successfully cleaved compared to that translated from the EF1-GFP-2A-mCher transgene. It is possible that inclusion of an additional amino acid spacer between the furin site and the GSG linker-2A site would improve cleavage efficiency of our transgenes. We have not examined this possibility. Addition of a furin cleavage site upstream of the 2A peptide, the cleavage of which would effectively remove the 2A peptide sequence from the C-terminus of the protein encoded by the upstream cistron, has also been reported to improve the function of this protein [38]. To minimize disruption of function of the upstream protein we routinely place cistrons that encode proteins amenable to C-terminus modifications upstream of the 2A peptide sequence in our vectors. Our and other investigators experiences with 2A sequences clearly indicate that it is important to examine the performance of each 2A construct in the cellular context in which they will be used.

In addition to the 2A transgenes examined in this study, we also examined the expression characteristics of bicistronic transgenes that were constructed using two independent EF1 promoters. Our decision to construct and examine the expression characteristics of the pFIN-EF1-GFP-EF1-mCher-WPRE vector in this study was prompted by the results of a previous study in which we found that the expression levels of two proteins encoded by dual-promoter vectors carrying two identical copies of a single photoreceptor-specific promoter, while not equal, were much higher than those obtained from IRES-containing transgenes [16]. The results of our analyses of the expression of pFIN-EF1-GFP-EF1-mCher-WPRE lentivirus in transduced HEK 293FT cells (Figure 2A–D) and in retinas (Figure 3A–F) were surprising because very little to no GFP expression was detected in infected cells. In retina, the GFP that was detected was not co-localized with mCher. Interestingly, inclusion of an additional WPRE element immediately after the upstream cistron (EF1-GFP-WPRE-EF1-mCher) did not significantly improve expression of GFP in transduced cells (Figure 2E–H) or retinas (Figure 3G–L). In fact, in our hands this modification, which was previously reported to enhance expression of both cistrons in a dual-promoter lentivector carrying identical hSynapsin promoters [26], produced a lentivirus that expressed only one or the other cistron in the vast majority of the infected cells. This unique protein expression pattern appeared to be observed only when the vectors were delivered in viral form. Both reporter proteins were co-expressed in HEK 293FT cells transfected with either plasmid DNA. One possible explanation for this observation is suggested by the expression characteristics of two of our dual-promoter lentivectors. When the EF1 promoters (1446 bp) used in this study to construct the EF1-GFP-EF1-mCher-WPRE transgene are replaced with either two rhodopsin kinase (297 bp) or interphotoreceptor retinol binding protein (262 bp) promoters, the reporter proteins are co-expressed in the retinal photoreceptors infected by the lentiviruses [16]. While untested, it is possible that the size of the EF1 promoter, which is approximately 5 times larger than either of the photoreceptor promoters, increases the probability of deletion of one of the cistrons during either viral packaging or reverse transcription of the viral RNA genome in infected cells prior to integration via homologous recombination [40]–[42]. Although the results obtained from our EF1 dual-promoter lentiviruses were undesired in our experiments, exclusive expression of either cistron of a bicistronic vector in transduced cells may be useful in some experimental or therapeutic applications.

One of the major focuses of our research program is to develop therapies for human LCA1. The animal model that we use in our studies is the GUCY1*B chicken, which is currently the only animal model for this disease that models both the genotype and phenotype of human LCA1. We have previously demonstrated that GC1 corrective gene therapy is sufficient to restore function to photoreceptor cells and reverse blindness in these animals [15], a finding corroborated by the results of this study. The primary difference between the lentiviral vectors used in these two studies was in the design of the bicistronic GC1 transgenes they carried. The bicistronic transgene used in our previous study contained an IRES element. In the current study, this element was replaced with a 2A-like cleavage sequence. Both bicistronic vectors produced sufficient amounts of GC1 to restore function to GUCY1*B photoreceptors and visual behavior to treated animals, but only the 2A transgene consistently expressed both of the encoded proteins. Importantly, our results indicate that the proline residue retained on the N-terminus of GC1 following co-translational ‘cleavage’ of the 2A peptide does not negatively affect the activity of the GC1 protein encoded by the GFP-2A-GC1 transgene.

To date, we have not yet achieved permanent vision restoration in GUCY1*B animals using corrective gene therapy alone, a problem that plagues many investigators working to design therapies to treat aggressive inherited photoreceptor diseases caused by mutations in genes expressed in these cells [3], [43], [44]. In the current study, we noted that higher percentages of retinal transduction with our GC1 vector were positively correlated with the strength of the visual responses exhibited by the animal and the duration of the benefits of the treatment. Our successes using corrective GC1 therapy alone suggest that it might be possible to achieve life-long restoration of vision in our model system by simply increasing the number of photoreceptors expressing the GC1 transgene. The fraction of the photoreceptor cell population that must be treated to achieve this outcome is unknown, but it is likely that the spatial relationships of the treated cells to other treated and untreated cells in the retina will have an impact on this value. We are interested in determining this value since it would provide a useful benchmark for comparing the effectiveness of different treatment strategies. A second approach that may lead to life-long vision restoration in our model would be to combine delivery of GC1 to the photoreceptors with delivery of additional therapeutic molecules (e.g. neurotrophic or anti-apoptotic factors) to enhance survival of the photoreceptors. The results of this study show that we will be able to successfully deliver combination therapies to photoreceptors and other retinal cells using bicistronic transgenes carrying 2A-like cleavage sequences. Efforts are currently underway to determine if combination therapies can improve treatment outcomes in our model of LCA1.

In summary, we examined the expression of three different bicistronic lentivectors in HEK 293FT cells and in neural retina and found that the lentivectors carrying transgenes containing 2A-like cleavage peptides were the only ones that reliably expressed both of the proteins encoded by the transgene and expressed them in a highly reproducible manner among infected cells. Since we were able to restore vision in GUCY1*B chickens using the 2A vector encoding GC1, our laboratory has now begun to develop 2A vectors that will permit us to determine if combining GC1 delivery with delivery of additional therapeutic proteins can permanently restore vision to these animals. While we use lentivectors in our studies, 2A-like cleavage peptides should prove valuable in any therapeutic or biotechnological application requiring co-expression of multiple proteins in targeted cells.

## Methods

### Ethics Statement

This study was carried out in strict accordance with the recommendations in the Guide for the Care and Use of Laboratory Animals of the National Institutes of Health. All animal protocols were approved by the University of Florida Institutional Animal Care and Use Committee (Approval 201004563) and adhered to the policies outlined in the Guide for the Care and Use of Laboratory Animals.

### Construction of Vectors

#### pFIN-EF1-GFP-EF1-mCherH-WPRE

pFIN-WPRE [16] was digested with NheI. GFP was amplified using PCR and a sense primer containing NheI and BsiWI and an antisense primer containing MluI and NheI. The PCR product was ligated into pFIN-WPRE at the NheI site to create pFIN-GFP-WPRE. This vector was then linearized using NotI and the EF1 promoter, which was amplified using sense and antisense primers containing NotI, was ligated into pFIN-GFP-WPRE to create pFIN-EF1-GFP-WPRE. A second EF1 promoter was amplified using a sense primer containing BsiWI and an antisense primer containing PacI and MluI sites and was ligated into pFIN-EF1-GFP-WPRE using BsiWI and MluI. Finally, hemagglutinin (HA)-tagged mCherry (mCherH) was created using PCR primers containing PacI sites and was ligated into the linearized pFIN-EF1a-GFP-EF1-WPRE to create the final construct. The antisense primer included the HA tag that was fused in frame to mCher immediately upstream of the stop codon. It was necessary to tag mCher with an epitope to which antibodies exist since we and others [45] have found that the antibodies that are currently available for mCher fail to detect this protein on western blots.

#### pFIN-EF1-GFP-WPRE-EF1-mCherH-WPRE

pFIN-EF1-GFP-EF1-mCherH-WPRE was linearized using BsiWI. WPRE was amplified using sense and antisense primers containing BsiWI sites and WPRE was ligated into the linearized backbone to create pFIN-EF1-GFP-WPRE-EF1-mCherH-WPRE.

#### pFIN-EF1-GFP-2A-mCherH-WPRE

pFIN-EF1-GFP-WPRE [16] was digested with NheI to remove GFP. GFP-2A-mCherH was created using a three-step PCR strategy described by Symczak et al [46] that included the addition of a GSG linker sequence upstream of the PTV1 2A peptide sequence. The final set of primers used to amplify GFP-2A-mCherH contained NheI sites that permitted it to be ligated into the NheI site in the pFIN-EF1a-WPRE backbone to create the final vector.

#### pFIN-EF1-GFP-Furin-2A-mCher-WPRE

This vector was created using the same strategy as that used to construct pFIN-EF1-GFP-2A-mCherH-WPRE except that the primers used to amplify GFP included sequence encoding the furin cleavage site (RAKR) that was inserted in frame just upstream of the GSG linker.

#### pFIN-EF1-GFP-2A-GC1-WPRE

The cDNA encoding bovine GC1 was removed from pBSII SK+-GC1 [15] using SpeI and KpnI and was ligated into our pFIN-WPRE backbone that had been digested with SpeI and KpnI. EF1 was amplified using sense and antisense primers containing NotI sites and was ligated into the NotI site in pFIN-GC1-WPRE. The resulting vector, pFIN-EF1-GC1-WPRE, was then digested with NheI and StuI, the StuI site being located approximately 700bp downstream from the start codon of the GC1 open reading frame. GFP-2A-GC1 (5’ 700 bp) was amplified using the three-step PCR method. The final sense and antisense primers used to amplify the GFP-2A-GC1 (5’ 700 bp) fragment contained NheI and StuI sites, respectively. GFP-2A-GC1 was then ligated into pFIN-EF1-GC1-WPRE to create pFIN-EF1-GFP-2A-GC1-WPRE.

### Lentiviral Packaging

The lentiviral vectors were packaged into lentivirus pseudotyped using vesicular stomatitis virus G (VSV-G) glycoprotein using a three plasmid packaging system as previously described [20]. Viral titers were estimated using a Lenti-X qRT-PCR kit (Chemicon, Billerica, MA) and typically averaged 2×10^12^ viral genomes per ml.

### Cell Culture and *In Vitro* Transfection and Transduction

HEK 293FT cells were grown and maintained in Dulbecco's Modified Eagle high glucose media containing 4.5 mg/ml D-glucose, L-glutamine and 0.11 µg/ml sodium pyruvate (Invitrogen, Carlsbad, CA) to which was added 10% fetal calf serum, 50 U/ml penicillin G, 50 µg/ml streptomycin, and 500 µg/ml geneticin. The cells were transiently transfected with plasmid DNA using Superfect (Qiagen, Valencia, CA) according to the manufacturer's instructions. In brief, 200,000 cells were seeded per well of gelatin-coated 6 well plates 24 h prior to transfection and were transfected with a mixture of 2.0 µg of DNA and 10 µl of Superfect per well. The transfected cells were allowed to proliferate for 48 h. To analyze the performance of the vectors when delivered as viral particles, HEK 293FT cells were plated as described above and were exposed to approximately 2×10^9^ viral particles for 72 h prior to analysis. Transfected or transduced cells were either harvested for Western blot analyses in SDS sample buffer supplemented with protease inhibitor cocktail (Roche, Indianapolis, IN) or were examined directly using a Zeiss AxioCam MRm digital camera system (Carl Zeiss Microimaging, Inc.,Thornwood, NY). GFP was detected using a narrow-band GFP filter set 41020 (Chroma Technology Corp, Bellows Falls, VT) and mCher was detected using a Chroma custom filter set that consisted of an exciter ET572/35, an emitter ET632/60, and a beamsplitter (Chroma Technology Corp, Bellows Falls, VT). For comparison purposes the exposure times used to photograph each reporter were kept constant between constructs. Hoechst (Molecular Probes Inc., Eugene, OR) was added to live cells for 3 min before analysis to visualize nuclei.

### Viral Injections of Chicken Embryos

All eggs used in this study were obtained from our wild-type Rhode Island Red and our GUCY1*B breeding colonies that we maintain at the University of Florida. Lentiviruses were injected into chicken embryos (stage 8–10) on embryonic day 2 (E2) as previously described [15]. Approximately 345 nl of virus was injected into the anterior region of the developing embryonic neural tube. The treated eggs were then sealed and incubated to E20 at which time the embryos were either sacrificed and the retinas were harvested for analyses or were hatched using our previously described protocol [15].

### Behavioral Analyses

The optokinetic nystagmus (OKN) responses, which are reflexive visual responses driven primarily by visual stimuli processed by the peripheral regions of the retina, were evaluated once a week for each treated chicken using previously published methods [15]. Two high contrast, vertical square wave grating stimuli with spatial frequencies of 0.065 or 0.26 cycles·degree^−1^ (bar widths of 5 cm and 1.25 cm, respectively) were used to elicit the responses. The behaviors were recorded using a digital camera (Canon Vixia HF20 HD video camera; http://www.usa.canon.com). The recorded behaviors were analyzed using a zero-three point scoring system: zero, no OKN response; one, inconsistent or unidirectional responses to the lower spatial frequency grating; two, consistent bidirectional responses to the lower spatial frequency grating; three, consistent bidirectional responses to the higher spatial frequency grating. The scores of the OKN responses for the right and left eyes of each animal obtained using the 5 or 1.25 cm bar stimuli were averaged to obtain the OKN response score for each stimulus on each test day.

### Retinal Whole-Mounts and Immunohistochemistry

Retinal whole mounts and immunohistochemistry were carried out as previously described [20]. The GFP and mCher reporter proteins were detected using either native fluorescence or immunohistochemistry. GFP and mCher native fluorescence was detected in retinal whole mounts and sections using the appropriate fluorescence filters and documented using a Zeiss AxioCam MRm digital camera. For immunohistochemistry, retinal whole mounts or cryosections (10 µm) were probed with a polyclonal rabbit anti-GFP antibody (1∶2000, overnight, kindly provided by W. Clay Smith, University of Florida, Gainesville, FL) and counterstained with 4′,6-diamidino-2-phenylindole (DAPI) where indicated. The primary antibody was detected using Alexa Fluor 488 (1∶500, Invitrogen). Zeiss filter set 02 was used to visualize DAPI-stained cell nuclei. To visualize GFP expression in retinal whole-mounts, retinas were incubated with a biotinylated, anti-rabbit secondary antibody and were processed using a Vectastain ABC kit and a 3,3′-diaminobenzidine (DAB) substrate kit for peroxidase (Vector laboratories, Inc., Burlingame, CA). The percent transduction of the retinal whole mounts was determined using the analyze particles feature of ImageJ version 1.44 h.

### Western Blot

Western blot analyses of the proteins expressed in transfected or transduced HEK 293FT cells and in transduced retinas were performed as previously described [47]. In brief, protein samples (1 µg of total transfected cell lysate, 15 µg of total transduced cell lysate, or 30 µg retina lysate) were separated on precast, gradient 4–12% polyacrylamide gels and transferred to PVDF membranes using an i-Blot transfer apparatus according to the manufacturer's instructions (Invitrogen, Carlsbad, CA). The blots were prepared in quadruplicate permitting each blot to be probed with a single antibody. Antibodies recognizing GFP, 2A, hemagglutinin, GC1, or GAPDH were used to probe the blots. After blocking the membranes in 5% milk/Tris Buffered Saline (TBS) for 1 h, the blots were incubated with primary antibody overnight at 4°C with gentle shaking. After three 10 minute washes in TBS, blots were incubated in secondary antibody diluted 1∶5000 in 5% milk/TBS for two hours at room temperature. The secondary antibodies used in this study were anti-chicken (GeneTex, Irvine, CA), anti-rabbit and anti-mouse (Cell Signaling Technology, Inc., Danvers, MA), and anti-rat (Sigma-Aldrich, St. Louis, MO). The blots were then washed and signals were visualized with ECL (Amersham, UK). Films were scanned using a GS-800 calibrated densitometer (BioRad, Hercules, CA) and the signals were quantified using Scion Image software (Scion Corp., Frederick, MD). For each bicistronic transgene, the densities of the mCherH and GFP-2A protein bands detected in the samples were determined and then normalized to the highest mCherH or GFP-2A sample value, respectively, which was assigned a value of 1.0. The relative levels of GFP-2A and mCherH produced by the transgenes were compared using analyses of variance and Tukey post hoc tests when appropriate (SigmaStat v2.03, Aspire Software International, Ashburn, VA). Graphs were generated using GraphPad Prism 5.0 (GraphPad Software, Inc., La Jolla, CA).

### Primary Antibodies

The primary antibodies used in the western blot experiments were chicken anti-GFP (1∶3000, Abcam, Cambridge, MA, Western blot analysis), rabbit anti-2A (1∶2000, Millipore, Billerica, MA), rabbit anti-GC1 (1∶500, kind gift from Dr. A. Yamazaki), rat anti-hemagglutinin, High Affinity (1∶5000, Roche, Indianapolis, IN), and mouse anti-GAPDH (1∶5000, Encor, Gainesville, FL). Rabbit anti-GFP (1∶2000, kind gift from Dr. W. Clay Smith) was used to stain retinal whole mounts.

## Supporting Information

Figure S1 Cleavage of GFP-2A-mCher and GFP-furin-2A-mCher polypeptides in transfected HEK 293FT cells. **(A, B)** Diagrams of the EF1-GFP-2A-mCher and EF1-GFP-furin (F)-2A-mCher transgenes. **(C)** Western blot showing cleavage of EF1-GFP-2A-mCher (-F) and EF1-GFP-F-2A-mCher (+F) transgenes. The blot was probed with an antibody against GFP that recognized both the cleaved and uncleaved polypeptides. The amount of total protein loaded per lane was either 20 or 50 µg.
(TIF)

Video S1 Optokinetic behavior of 8-day-old wild-type chicken. The responses shown were elicited by a high-contrast vertical square wave grating with a spatial frequency of 0.26 cycles·degree^−1^ (bar width 1.25 cm). Reflexive head movements characteristic of the optokinetic response in birds were observed when the stimulus was rotated either clockwise or counter clockwise. No optokinetic responses were elicited by the white control stimulus
(MP4)

Video S2 Optokinetic behavior of 8-day-old untreated GUCY1*B chicken. No responses were elicited by either of the high-contrast vertical square wave grating stimuli. The stimuli used in this particular test were a high-contrast vertical square wave grating with a spatial frequency of 0.26 cycles·degree^−1^ (bar width 1.25 cm) and a solid white stimulus.
(MP4)

Video S3 Optokinetic behavior of 8-day-old GUCY1*B chicken that had been treated with pFIN-EF1-GFP-2A-GC1-WPRE lentivirus on embryonic day 2. This treated animal, unlike untreated animals, exhibited a robust, optokinetic response that was assigned a score of 3.0. Reflex responses were elicited by the high-contrast vertical square wave grating (bar width 1.25 cm) when rotated in either clockwise or counter clockwise. No responses were elicited by the solid white control stimulus.
(MP4)
