# Supplementary figures and images for: Bicistronic Lentiviruses Containing a Viral 2A Cleavage Sequence Reliably Co-Express Two Proteins and Restore Vision to an Animal Model of LCA1
Source: PLoS One. 2011 May 27;6(5):e20553. doi: 10.1371/journal.pone.0020553 (PMC3103589; doi:10.1371/journal.pone.0020553)

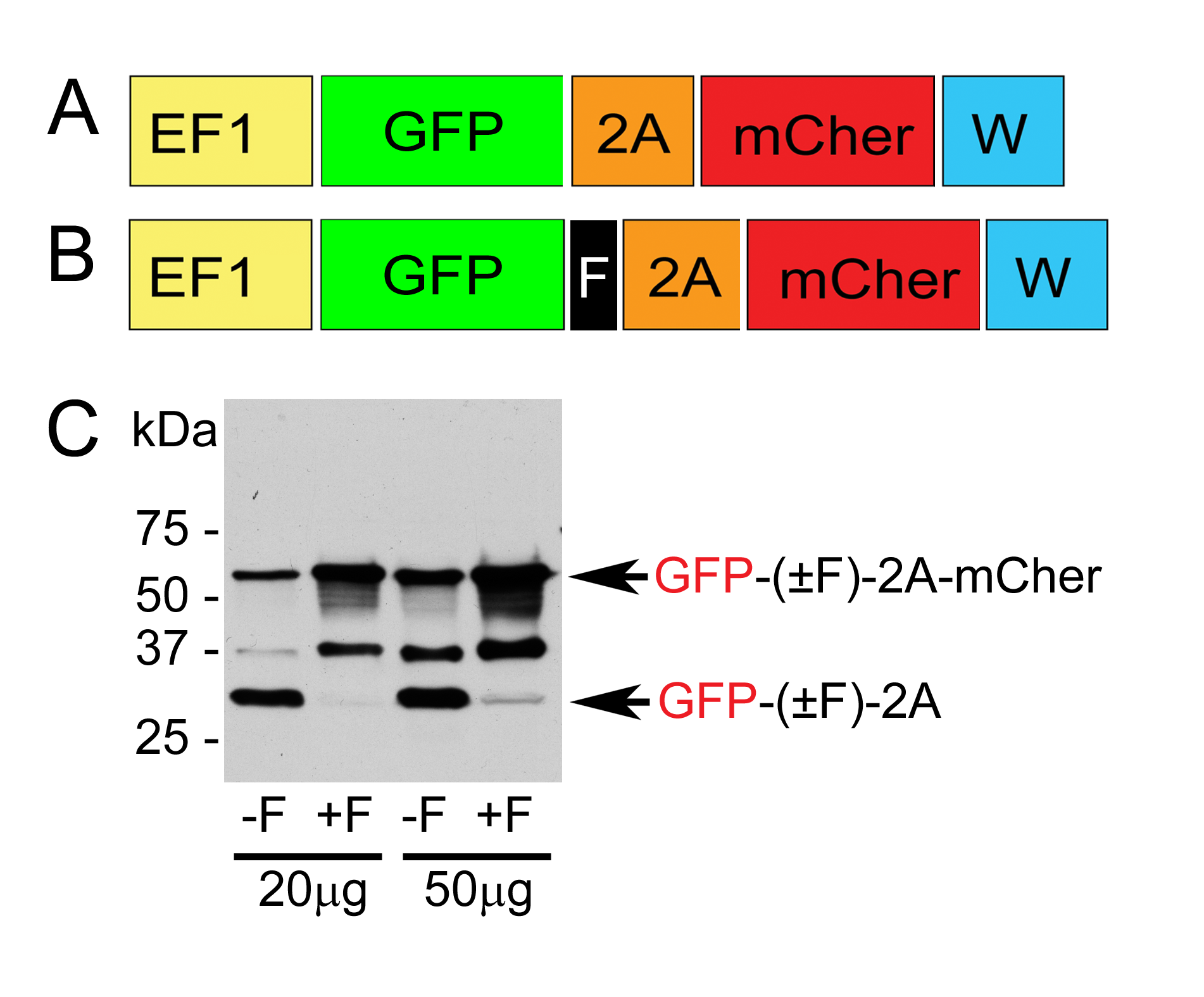

Supplement: Figure S1 — Cleavage of GFP-2A-mCher and GFP-furin-2A-mCher polypeptides in transfected HEK 293FT cells. (A, B) Diagrams of the EF1-GFP-2A-mCher and EF1-GFP-furin (F)-2A-mCher transgenes. (C) Western blot showing cleavage of EF1-GFP-2A-mCher (-F) and EF1-GFP-F-2A-mCher (+F) transgenes. The blot was probed with an antibody against GFP that recognized both the cleaved and uncleaved polypeptides. The amount of total protein loaded per lane was either 20 or 50 µg. (TIF) [file pone.0020553.s001.tif]
